# Gender differences in biochemical characteristics and health-related quality of life among hemodialysis patients

**DOI:** 10.1186/s12882-026-04867-4

**Published:** 2026-03-21

**Authors:** Lamis Khedr, Aber Halim, Ahmed Emara, Badry Talaat, Mohamed Ali

**Affiliations:** Nephrology Department, Ain Shams University, Cairo, Egypt

**Keywords:** Hemodialysis, Gender differences, Developing world, KDQOL

## Abstract

**Background:**

Women receiving hemodialysis face a range of social and medical challenges that can significantly affect their treatment and overall quality of life. We aimed to assess whether there are gender differences in hemodialysis by comparing biochemical characteristics and dialysis parameters between males and females. We used Kidney Disease Quality of Life Short Form (KDQOL-SF™ 1.3) questionnaire to compare health- related quality of life.

**Methods:**

A cross-sectional study was conducted involving 340 prevalent hemodialysis patients from three dialysis centres. Data were collected between August 2024 and February 2025. Sequential general linear models (GLM) were applied to assess gender differences in Physical component summary scores and Mental component summary scores: Model 1 (unadjusted), Model 2 (adjusted for physiological variables: age, haemoglobin, albumin, PTH, and Kt/V), and Model 3 (further adjusted for social variables: marital status, education, and employment). Statistical significance was set at *p* < 0.05.

**Results:**

Haemoglobin levels were similar in both genders with a mean of 9.2 g/dL. Serum calcium (8.6 vs. 8.4 mg/dL, *p* = 0.25) phosphate levels (5.1 vs. 4.9 mg/d, *p* = 0.3) were similar. Parathyroid hormone was higher in females compared to males (589 ± 551 pg/mL vs. 744 ± 708 pg/mL, *p* = 0.026). There was no difference in dialysis adequacy, KT/V (1.55 ± 1.1 vs. 1.54 ± 0.9, *p* = 0.4). Vascular access type was similar, AVF (89.0% vs. 87.9%, *p* = 0.95). There was no difference in PCS (29.11 ± 6.75 vs. 28.93 ± 6.53, *p* = 0.807). In contrast, Mental component summary was higher in males (45.05 ± 5.98 vs. 41.07 ± 6.01; *p* < 0.001). After adjustment for all covariates, gender remained a strong independent predictor of Mental component summary, with females scoring significantly lower than males (B = − 3.90, 95% CI [–5.24, − 2.57], *p* < 0.001).

**Conclusion:**

In this study there were no significant gender differences in biochemical characteristics or dialysis adequacy parameters. Mental component summary was significantly lower in female patients, and this difference remained after adjustment for physiological and social covariates emphasizing the need for gender-sensitive, psychosocially informed care models.

## Background

Women receiving hemodialysis face a range of social and medical challenges that can significantly affect their treatment and overall quality of life. In developing countries, cultural expectations and family caregiving responsibilities often delay their presentation to dialysis services and later affect adherence to hemodialysis sessions [1]. In addition, they can depend on family members for financial assistance and transportation to dialysis units. Some studies in developing counties have reported that women receive fewer vascular access surgeries for permanent fistula creation compared with men [2]. Other studies even reported that women on dialysis also have a higher risk of morbidity, hospitalization, and dialysis withdrawal than their male counterparts [3, 4]. These factors contribute to their lower reported health-related quality of life. The commencement of dialysis is a life changing event that affects employment and relations owing to the 12-hr week commitment to dialysis and the challenges that might be faced at the beginning of treatment including access problems and discomfort during dialysis. Patients may begin dialysis with either low or high expectations influenced by the experiences of friends or family members. Research has shown that women often score lower in the Physical Component Summary, which includes physical functioning, role limitation due to physical health, general health, and bodily pain. Similarly, scores in the Mental Component Summary—covering emotional well-being, social functioning, vitality, and role limitations due to emotional problems—are consistently lower among female dialysis patients in developing countries [5].

## Aim

We aimed to assess gender differences in health-related quality of life in a cohort of hemodialysis patients and to explore whether disparities in mental health–related quality of life could be explained by differences in dialysis adequacy, biochemical parameters, or socioeconomic characteristics.

### Methods

**Study design** A cross-sectional study was conducted involving 340 prevalent hemodialysis patients from three dialysis centres in Cairo, Egypt. Data were collected between August 2024 and February 2025.

**Study participants** All participants over the age of 18 were included in this study. Patients who were excluded included incident haemodialysis patients, patients with malignancy, active infection or requiring blood transfusion and patients with missing records or refusing to participate.

### Data collection

Demographic and clinical data, including age, aetiology of kidney disease, body mass index (BMI), education and employment status were obtained at enrolment. Written informed consent was obtained from all participants after the study objectives were clearly explained. Questionnaires were distributed by nephrology physicians. Routine laboratory investigations, dialysis prescription details were extracted from medical records at the time of enrolment. Patients were assigned a study number. 

**Fig. 1:**
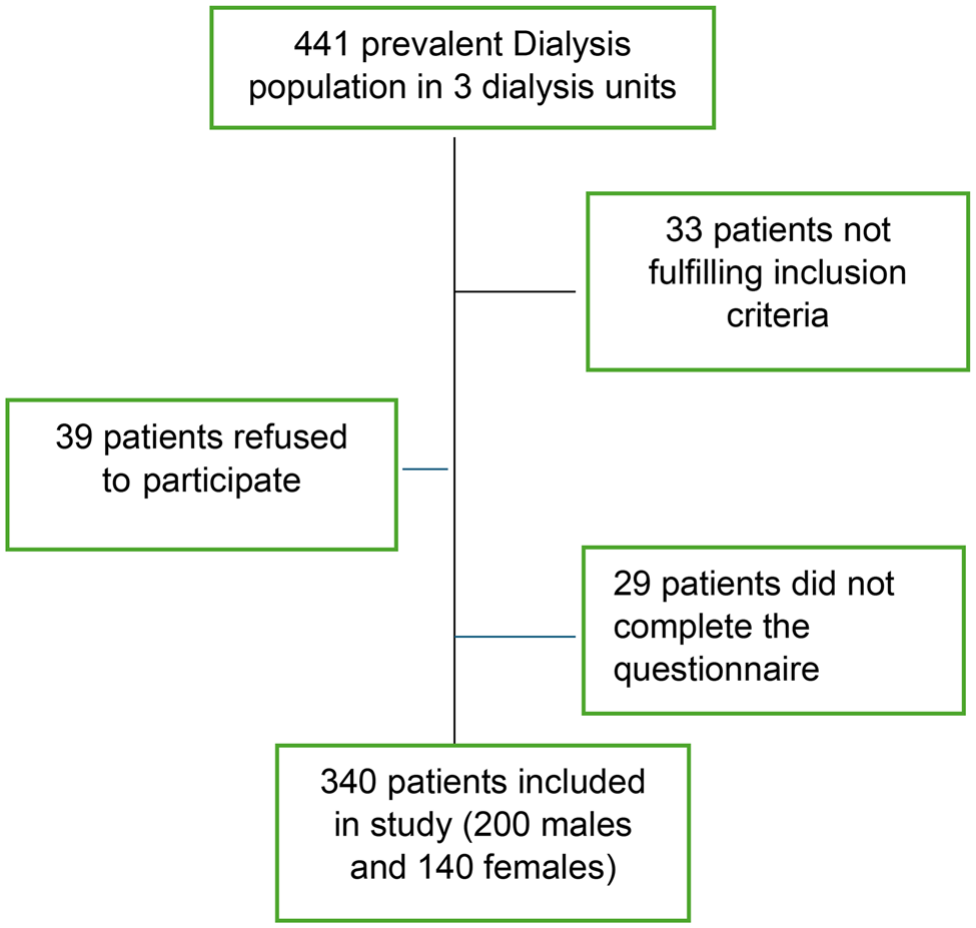
Flow chart of the study participants

A total of 441 patients were receiving maintenance hemodialysis at the participating Ain Shams University–affiliated dialysis units at the time of the study. Of these, 408 patients met the inclusion criteria. Thirty-nine patients declined participation, and twenty-nine returned incomplete questionnaires as shown in Fig. 1. Consequently, 340 participants (200 males and 140 females) were included in the final analysis. The overall survey completion rate was 91%, indicating high participant engagement and data completeness.

### Questionnaire

The disease-specific instrument used in this study was the Kidney Disease Quality of Life-Short Form (KDQOL-SF™) version 1.3, a self-report measure developed for individuals who have kidney disease and are on dialysis. We used the Arabic translated version KDQOL-SF™ 1.3 questionnaire. Version 1.3, which assesses quality of life in kidney failure patients who have kidney disease and are on dialysis [6]. Participants completed paper-based, self-administered questionnaires. The KDQOL-SF™ 1.3 includes 36 general health items and 43 kidney disease–specific items. The general health section covers eight subscales-Physical Functioning, Role Physical, Pain, General Health, Emotional Well-Being, Role Emotional, Social Functioning, and Energy/Fatigue in addition to one overall health item (Table 1). Scores for all domains were calculated according to the user manual, the resulting scores from 0 to 100, with higher scores indicating better quality of life [7].

**Table 1:** KDQOL-SF™

Scale	Number of items
**ESRD Targeted area**	43
Symptom/Problem of kidney disease (SPKD)	12
Effect of Kidney disease (EKD)	8
Burden of Kidney disease (BKD)	4
Work status	2
Cognitive function	3
Social interaction	3
Sexual function	2
Sleep	4
Social support	2
Dialysis staff encouragement	2
Patient Satisfaction	1
**36 items of health survey (SF-36)**	
Physical functioning	10
Role Physical Limitation	4
Pain	2
General Health	5
Emotional well-being	5
Role emotional limitation	3
Social function	2
Energy/Fatigue	4

KDQOL-SF™ 1.3 subscales and their item composition adapted from RAND Corporation. Kidney Disease Quality of Life Short Form (KDQOL-SF™) Version 1.3: A Manual for Use and Scoring. Santa Monica, CA; 1995 [7]

### Subscales

Physical functioning evaluates how much a person’s current health affects their ability to perform everyday physical tasks, such as walking various distances, climbing stairs, carrying items like groceries, or completing basic self-care activities like bathing and dressing. Role physical limitation measures how much a physical health problems usual work, daily tasks, social roles or responsibilities. Emotional well-being reflects general mood including feelings of happiness, anxiety and depression. Role emotional limitation reflects limitations in work or activities due to emotional problems. Social function defines the level of social life limitations caused by physical and emotional discomfort [8].

### Ethical consideration

This study was performed in accordance with the ethical standards of Ain Shams University Research Committee and with the 1964 Helsinki declaration and its later amendments or comparable ethical standards. Ain Shams University Faculty of Medicine Research Ethics Committee (REC) FWA 000017585 has approved the study protocol- patients gave their written consent to participate.

### Statistical analysis

Statistical analyses were performed using SPSS version 29.0. Continuous data were presented as mean ± SD and categorical data as frequencies and percentages. Between-gender comparisons used independent samples *t*-tests or Chi-square tests. Sequential general linear models (GLM) were applied to assess gender differences in PCS and MCS scores: Model 1 (unadjusted), Model 2 (adjusted for physiological variables: age, haemoglobin, albumin, PTH, and Kt/V), and Model 3 (further adjusted for social variables: marital status, education, and employment). Statistical significance was set at *p* < 0.05.

## Results

### Baseline characteristics

A total of 340 participants were included (140 females, 200 males). Mean age was similar between males (47.8 ± 16.1 years) and females (46.5 ± 15.8 years). All females were non-smokers, while 21% of males were current or former smokers. Females showed a non-significant trend toward higher employment (61.4% vs. 50.5%; *p* = 0.14) and higher educational attainment (72.1% vs. 64.5%; *p* = 0.14). Hypertension was the most common comorbidity in both groups. Vascular access was comparable, with arteriovenous fistulas used in 89.0% of males and 87.9% of females (*p* = 0.95) as shown in Table 2.

**Table 2:** Baseline characteristics of the study participants

	Male (*n* = 200)	Female (*n* = 140)	*P* value
Age, years (mean ± SD)	47.8 ± 16.1	46.5 ± 15.8	0.40¹
BMI (mean ± SD)	26.0 ± 5.4	25.7 ± 5.0	0.09¹
Smoking status, n (%)			< 0.001²*
• Current smoker	9 (4.5)	0 (0)	
• Ex-smoker	33 (16.6)	1 (0.7)	
Marital status, n (%)			0.03²*
• Married	165 (81.5)	101 (72.1)	
• Other (single, divorced, widowed)	35 (18.5)	39 (27.9)	
Employment status, n (%)			0.14²
• Part time Employed	101 (50.5)	86 (61.4)	
• Unemployed	92 (46.0)	50 (35.7)	
• Student	7 (3.5)	4 (2.9)	
Education, n (%)			0.14²
• Primary education	71 (35.5)	39 (27.9)	
• Higher education	129 (64.5)	101 (72.1)	
Cause of kidney failure, n (%)			0.12²
• Hypertension	83 (41.5)	40 (28.6)	
• Diabetes	17 (8.5)	19 (13.6)	
• Unknown	49 (24.5)	32 (22.9)	
• Chronic pyelonephritis	15 (7.5)	8 (5.7)	
• Analgesic overuse	9 (4.5)	8 (5.7)	
• Glomerulonephritis	9 (4.5)	9 (6.4)	
• ADPKD	6 (3.0)	3 (2.1)	
• Lupus nephritis	1(0.5)	10 (7.2)	
•Preeclampsia	0 (0.0)	6 (4.3)	
•Others	11(5.5)	5(3.6)	
Vascular access n, % AVF Permcath CVC	178(89.0) 17(8.5) 5(2.5)	123(87.9) 13(9.3) 4(2.9)	0.95²
Vascular access complications, n (%)	178 (89)	124 (88.6)	0.47²

¹ Independent samples t-test used for continuous variables. ² Chi-square test used for categorical variables Statistically significant at *P* < 0.05*.BMI: Body mass index, AVF: Arteriovenous fistula, CVC: Central venous catheter

### Dialysis treatment parameters

In Table 3 Dialysis parameters and anticoagulation methods showed no significant gender differences. The most common blood flow rate was 300–400 mL/min in both groups. Ultrafiltration rates were similar in males and females (10.7 vs. 10.3 mL/kg/hr). Intradialytic complications were comparable, including itching (33.5% vs. 31%), hypotension (15.5% vs. 20%), cramps (5% vs. 3.6%), and hypertension (3% vs. 1.4%) in males and females, respectively. Compliance with dialysis was high and similar between groups (89.0% vs. 88.6%; *p* = 0.68), as was the rate of recurrent hospitalization (13.5% vs. 10.7%; *p* = 0.44).Dialysis adequacy, assessed using Kt/V determined by single-pool urea kinetic modelling, was 1.4 in males and 1.5 in females. Predialysis and post dialysis urea samples were obtained from the same hemodialysis session. Kt/V was calculated using the Daugirdas et al. formula [9]:

$$\begin{aligned}\rm Kt/V=&-ln (Ratio-0.03)+ [(4-(3.5 \times Ratio))\cr&\times (UF / Wt.)]\end{aligned}$$ where Ratio is post-BUN/pre-BUN, UF is ultrafiltration volume, and Wt is post-dialysis weight.

Non-compliance was defined as missing more than three dialysis sessions per month without a medical reason or consistently shortening session duration. Recurrent hospitalization was defined as more than two hospital admissions in the past 6 months for causes other than vascular access problems.

**Table 3:** Dialysis treatment parameters of the study participants

Dialysis vintage, months (mean ± SD)	14.81 ± 6.05	14.07 ± 5.2	0.006*
Time for allocation of dialysis slot in months, n (%) < 3 3–6	80(40) 120(60.0)	51(36.5) 89(63.5)	0.20²
Dialysis frequency, n (%)			0.50²
• 3 per week	190 (95)	135 (96.4)	
• 2 per week	10 (5)	5 (3.6)	
Blood flow rate, ml/min			0.20¹
• 250–300	56 (28.0)	54 (38.5)	
• 300–400	144 (72.0)	86 (61.5)	
UF rate, ml/kg/hr (mean ± SD)	10.7 ± 4.5	10.3 ± 4.3	0.40¹
Kt/v (mean ± SD)	1.55 ± 1.1	1.54 ± 0.9	0.40¹
Weekly epoetin alpha dose, IU			0.40²
• <4000	4 (2.5)	3 (2.1)	
• 4000–8000	121 (60.5)	82 (58.5)	
• >8000	75 (37.0)	55 (39.4)	
Anticoagulation, n (%)			0.50²
• Heparin	188 (94.0)	133 (95.0)	
• Clexane	3 (1.5)	2 (1.4)	
• Heparin free	9 (4.5)	5 (3.6)	
Dialysis complications, n (%)			0.68²
• No complications	86 (43.0)	61 (43.6)	
• Hypotension	31 (15.5)	28 (20.0)	
• Cramps	10 (5.0)	5 (3.6)	
• Itching	67 (33.5)	44 (31)	
• Hypertension	6 (3)	2 (1.4)	
Compliance, n (%)	178 (89)	124 (88.6)	0.68²
Recurrent hospitalization, n (%)	27 (13.5)	15 (10.7)	0.44²

¹ Independent samples t-test used for continuous variables. ² Chi-square test used for categorical variables Statistically significant at *P* < 0.05*. UF rate: Ultrafiltration rate

### Biochemical characteristics

Haemoglobin levels were similar in both groups (mean 9.2 g/dL). Ferritin, transferrin saturation (TSAT), and total iron-binding capacity (TIBC) were also similar. There were no statistically significant gender differences in serum calcium (8.6 vs. 8.4 mg/dL, *p* = 0.25) or phosphate levels (5.1 vs. 4.9 mg/dl, *p* = 0.3). PTH concentrations were significantly higher in females compared to males (589 ± 551 vs. 744 ± 708, *p* = 0.026) as shown in Table 4.

**Table 4:**
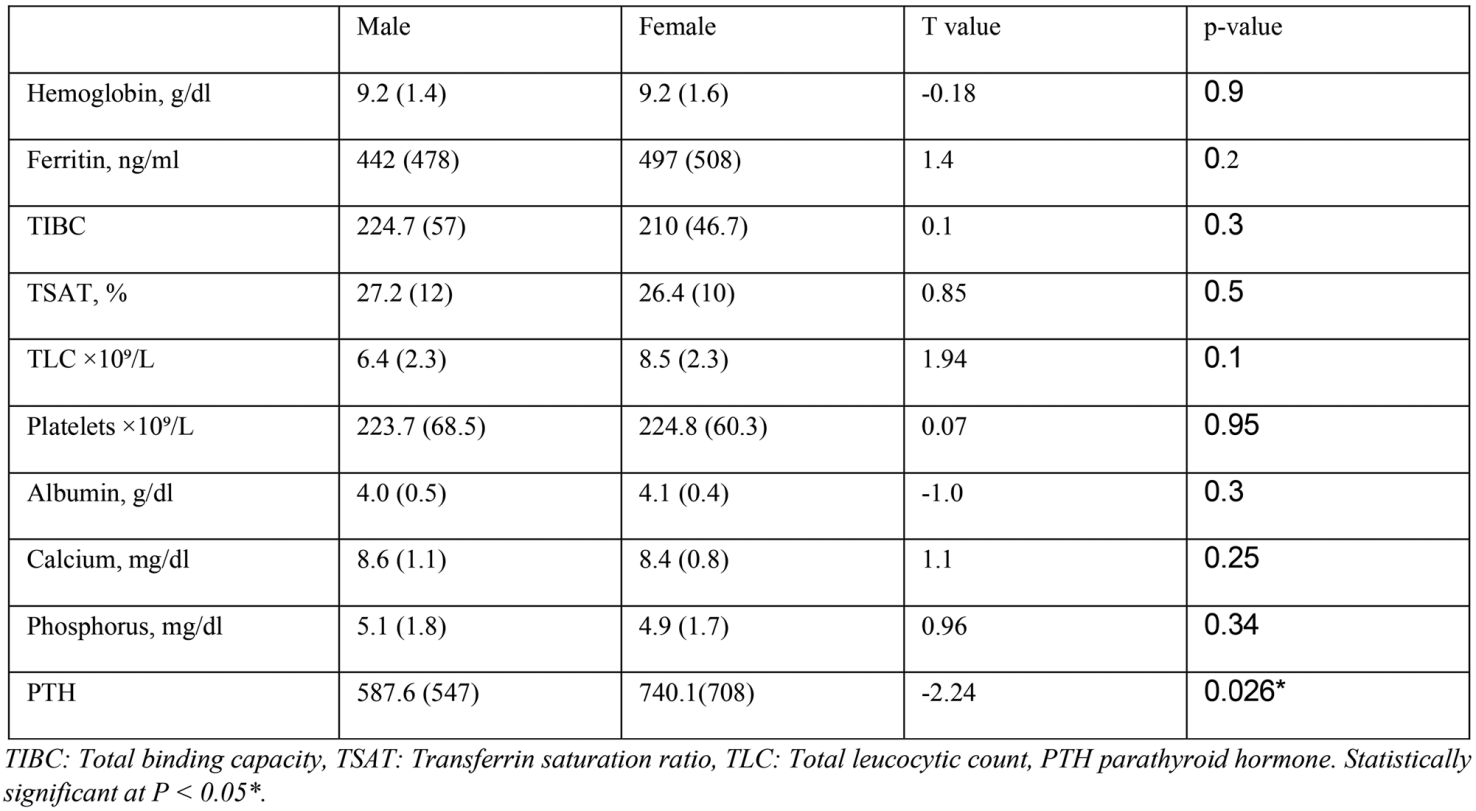
Biochemical characteristics of the study participants

### Kidney Disease Quality of Life

#### ESRD targeted domains

Table 5 summarizes the comparison of ESRD targeted areas including the Symptoms and Problems of Kidney Disease (SPKD), Effects of Kidney Disease (EKD), Burden of Kidney Disease (BKD), Cognitive function, Social interaction, Sexual function, Sleep and Social support between male and female participants. The mean SPKD scores were comparable between males (52.3 ± 16.14) and females (51.4 ± 14.9). Similarly, the mean EKD scores were (61.8 ± 14.6) for males and (61.5 ± 13.6) for females, as well as BKD scores (27.75 ± 18.76) for males and (29.17 ± 19.57). Independent-samples *t*-tests found no statistically significant gender differences across any of the KDQOL domains, (*p* > 0.05). Sexual Function domain approached significance (*p* = 0.076), with males reporting slightly higher scores.

#### 36 items of health survey

Males demonstrated significantly higher physical functioning scores than females (30.4 ± 17.7 vs. 24.4 ± 16.0, *p* = 0.002). The analysis also revealed a significant gender difference in emotional well-being scores. Males reported a higher mean score (56.8 ± 11.05) compared to females (43.0 ± 11.9), with a wide range of scores observed in both groups. The independent samples t-test indicated a statistically significant difference between the two groups (t = 10.9, *p* < 0.001). Pain scores are very similar for males and females as well as role emotional scores and social function (*p* > 0.05). The comparison of energy scores between males and females revealed a statistically significant difference. Males reported a higher mean energy score (41.5 ± 19.2) compared to females (31.5 ± 16.5), respectively (t = 5.0, *p* < 0.001), suggesting that males tend to experience greater energy levels than females in this sample.

**Table 5:** Comparison of KDQOL domains between study participants

	Male (*n* = 200)	Female (*n* = 140)	t	*p*
SPKD	52.30 (16.14)	51.41(14.92)	0.52	0.605
EKD	61.83 (14.63)	61.48 (13.58)	0.23	0.819
BKD	27.75 (18.76)	29.17 (19.57)	−0.67	0.501
Cognitive Function	64.93 (14.41)	65.23 (16.73)	−0.18	0.083
Social Interaction	68.81 (16.46)	69.36 (16.65)	−0.30	0.765
Sexual Function	78.43 (21.71)	74.01 (23.84)	1.78	0.076
Sleep	61.76 (45.98)	61.71 (29.51)	0.01	0.992
Social Support	55.09 (26.06)	56.72 (22.71)	−0.60	0.551
Physical functioning	30.4 (17.7)	24.4 (16.0)	3.00	0.002*
Role physical limitation	24.1(22.0)	23.7(26)	0.15	0.9
Pain	45.3 (20.8)	43.5 (23.6)	0.7	0.5
General Health	39.8 (13.8)	37.5 (13.5)	1.52	0.13
Emotional well-being	56.8 (11.05)	43(11.9)	10.9	<0.001*
Role emotional limitation	65.0 (27.8)	66.0 (26.4)	-0.44	0.66
Social function	43.1(19.4)	43.0 (18.6)	0.04	0.97
Energy	41.5 (19.2)	31.5 (16.5)	5	<0.001*

Values are means (standard deviations). Higher scores indicate better quality of life. t = independent-samples t-test statistic; Statistically significant at *P* < 0.05*

As shown in Table 6, there was no significant difference in physical component summary between males and females (PCS) (29.11 ± 6.75 vs. 28.93 ± 6.53; *p* = 0.807).In contrast, mental component scores were significantly higher in males (MCS)(45.05 ± 5.98) compared with females (41.07 ± 6.01; *p* < 0.001), indicating better emotional well-being and social functioning among male participants.

**Table 6:** Comparison of PCS and MCS between the study participants

Domain	Male (Mean ± SD)	Female (Mean ± SD)	Mean Difference (95% CI)	t	*P*
PCS	29.11 ± 6.75	28.93 ± 6.53	0.18 (–1.26 to 1.62)	0.25	0.807
MCS	45.05 ± 5.98	41.07 ± 6.01	3.98 (2.68 to 5.28)	6.03	< 0.001*

Physical (PCS) and Mental (MCS) Component Summary scores. Values are expressed as mean ± standard deviation. p values obtained from independent-samples t-tests. CI = confidence interval. Statistically significant at *P* < 0.05*

Table 7 summarizes the effects of gender on PCS and MCS scores across sequential models. In Model 2 after adjusting for physiological covariates (age, haemoglobin, serum albumin, parathyroid hormone, and Kt/V), no significant gender differences were found in the PCS scores. Among the physiological variables, haemoglobin was the only variable showing an association with PCS (*p* = 0.049). Age, albumin, PTH, and Kt/V were not significantly associated with PCS scores. MCS score was statistically significant (F (6, 333) = 6.44, *p* < 0.001, Adjusted R² = 0.088). After adjustment for physiological factors, females scored significantly lower on MCS than male patients (B = − 3.90, 95% CI [–5.21, − 2.59], *p* < 0.001). None of the physiological covariates were associated with MCS. In Model 3, the fully adjusted general linear models controlling for both physiological and social variables (marital status, employment, education, and gender), no statistically significant associations were found with the PCS score. The overall model was not significant (F (9, 330) = 1.07, *p* = 0.383). Haemoglobin showed a borderline positive association with PCS (*p* = 0.055), however this did not reach statistical significance. MCS was statistically significant (F (9, 330) = 4.26, *p* < 0.001), explaining approximately 10% of the variance (Adjusted R² = 0.080). After adjustment for all covariates, gender remained a strong independent predictor of MCS, with females scoring significantly lower than males (B = − 3.90, 95% CI [–5.24, − 2.57], *p* < 0.001). None of the physiological or social covariates showed significant associations with MCS.

**Table 7:** Comparison of PCS and Mental MCS by gender across three models

Outcome	Model	Male Mean ± SD	Female Mean ± SD	Mean Difference (95% CI)	*p*	Model Significance (F, df, *p*)	Adjusted *R*²
PCS	Model 1	29.11 ± 6.75	28.93 ± 6.53	0.18 (–1.26 to 1.62)	0.807	F(1, 338) = 0.25, *p* = 0.807	–0.003
	Model 2	—	—	–0.20 (–1.65 to 1.26)	0.791	F(6, 333) = 1.35, *p* = 0.236	0.006
	Model 3	—	—	–0.19 (–1.66 to 1.28)	0.799	F(9, 330) = 1.07, *p* = 0.383	0.002
MCS	Model 1	45.05 ± 5.98	41.07 ± 6.01	3.98 (2.68 to 5.28)	< 0.001	F(1, 338) = 6.03, *p* < 0.001*	0.100
	Model 2	—	—	–3.90 (–5.21 to − 2.59)	< 0.001	F(6, 333) = 6.44, *p* < 0.001*	0.088
	Model 3	—	—	–3.90 (–5.24 to − 2.57)	< 0.001	F(9, 330) = 4.26, *p* < 0.001*	0.080

Model 1 = unadjusted (independent-samples t test); Model 2 = adjusted for physiological covariates (Age, Haemoglobin, Albumin, PTH, Kt/V); Model 3 = adjusted for physiological + social covariates (Marital status, Employment, Education, Gender)

Values represent mean ± standard deviation for males and females, mean differences with 95% confidence intervals, and model statistics. Statistically significant at *P* < 0.05*

In Table 8 summary scores after adjusting for all covariates, no significant predictors of PCS were identified. The overall PCS model was not significant (F (9, 330) = 1.07, *p* = 0.38) with a very low power (Adjusted R² = 0.002). Among all predictors, haemoglobin showed a weak positive association (B = 0.48, 95% CI − 0.01 to 0.97, *p* = 0.06), suggesting that higher haemoglobin levels might be linked with slightly better physical functioning, but this did not reach statistical significance. In contrast, the MCS model remained statistically significant (F (9, 330) = 4.26, *p* < 0.001). Gender continued to be the only significant factor after full adjustment, with females scoring approximately 3.9 points lower than males (B = − 3.90, 95% CI − 5.24 to − 2.57, *p* < 0.001). None of the other covariates were associated with MCS.

**Table 8:** Fully adjusted model for Physical (PCS) and Mental (MCS) Component Summary scores

Predictor	PCS B (95% CI)	*p*	MCS B (95% CI)	*p*
Age	–0.03 (–0.08 to 0.01)	0.15	+ 0.01 (–0.03 to 0.05)	0.64
Haemoglobin	+ 0.48 (–0.01 to 0.97)	0.06	–0.15 (–0.60 to 0.30)	0.51
Albumin	–1.13 (–2.79 to 0.52)	0.18	–0.81 (–2.31 to 0.69)	0.29
PTH	0.00 (–0.00 to 0.00)	0.73	0.00 (–0.00 to 0.00)	0.79
Kt/V	–0.27 (–0.99 to 0.45)	0.47	+ 0.20 (–0.45 to 0.86)	0.55
Marital status	–0.44 (–2.18 to 1.30)	0.62	+ 0.05 (–1.53 to 1.63)	0.95
Employment	–0.79 (–2.11 to 0.53)	0.24	+ 0.16 (–1.04 to 1.36)	0.80
Education	+ 0.16 (–1.43 to 1.75)	0.84	–0.05 (–1.49 to 1.40)	0.95
Gender (Female vs. Male)	–0.19 (–1.66 to 1.28)	0.80	–3.90 (–5.24 to − 2.57)	< 0.001*

Fully adjusted general linear models for Physical (PCS) and Mental (MCS) Component Summary scores controlling for physiological (age, haemoglobin, albumin, PTH, Kt/V) and social (marital status, employment, education, and gender) variables

Values represent unstandardized regression coefficients (B) with 95% confidence intervals and p values. Statistically significant at *P* < 0.05*

## Discussion

Hemodialysis is the main renal replacement modality in the Egyptian CKD population. There are approximately 70,000 dialysis patients in Egypt in 2024 with annual increase of 10% constituting 0.06% of the population [10]. This study was conducted in three dialysis centres affiliated to one of the largest university hospitals in Cairo. We used the Arabic version of the KDQOL-SF1.3 questionnaire, which is a valid and reliable tool for use in Egyptian patients with CKD and those on dialysis [11].

Female patients demonstrated higher levels of educational attainment and employment compared with males, although employment was predominantly part-time. This finding likely reflects the urban setting of the study, where educational and occupational opportunities for women are relatively greater than in rural areas of Egypt [12].

### Clinical and biochemical characteristics

Anaemia was prevalent, with an average haemoglobin level of 9.2 g/dl. There was no difference between males and females, but both were markedly lower than the levels recommended by the KDIGO guidelines for anaemia management in ESRD [13]. This is likely related to underutilization of erythropoiesis-stimulating agents reflecting partial governmental subsidy caps [14]. Ferritin and TSAT values were comparable between the two groups and represented averages from peer reviewed studies [15]. Parathyroid hormone (PTH) levels were markedly elevated in both groups, particularly among females, exceeding KDIGO-recommended targets for CKD–mineral and bone disorder (CKD-MBD) [16]. These findings agreed with Indridason et al., who reported higher PTH in female patients [17], though other studies report mixed results [18, 19]. Serum calcium levels were within recommended range, while phosphate levels showed better control among females. Persistent hyperphosphatemia remains a major challenge in ESRD management and is associated with secondary hyperparathyroidism, vascular calcification, and adverse cardiovascular outcomes [20, 21].

### Equity in dialysis care delivery

This study demonstrated no evidence of gender bias in the delivery of dialysis care. Time to dialysis allocation, vascular access type, and dialysis adequacy were comparable between males and females. The proportion of patients using arteriovenous fistulas was similar across sexes and comparable to rates reported from dialysis centres in developed countries [22, 23]. This contrasts with reports from other low- and middle-income settings, such as India, where gender disparities in dialysis access have been documented [2]. Dialysis adequacy, assessed by Kt/V, was generally within KDOQI-recommended targets [24], with slightly higher values observed in women, likely reflecting sex-related differences in body composition.

### Gender differences in health-related quality of life

The mean Physical Component Summary (PCS) was comparable between males and females. The mean Mental Component Summary (MCS) score was lower in females, with a difference of 4 points compared with males. The association between female gender and lower mental health–related quality of life persisted after adjustment for age, key biochemical markers (haemoglobin, albumin, parathyroid hormone), dialysis adequacy (Kt/V) and core sociodemographic variables including employment status, education, and marital status. This adjustment suggests that the observed disparity in Mental Component Summary scores is unlikely to be explained by differences in disease severity, dialysis quality, or conventional socioeconomic factors. Rather, the persistence of this disparity points towards the influence of unmeasured gender-related psychosocial determinants.

Although biological sex-related factors, such as hormonal changes or menopause, may influence symptom perception, the persistence of lower MCS scores after extensive adjustment strongly suggests a dominant role for gender-related psychosocial determinants. A clear distinction between biological sex effects and gender-related influences is therefore essential when interpreting HRQOL outcomes in dialysis populations. Biological sex effects primarily reflect physiological differences, whereas gender encompasses socially constructed roles, expectations, and lived experiences that shape mental well-being.

### Sociocultural context and mental health

In the Egyptian and broader Middle Eastern context, women often face greater social constraints and carry a larger share of caregiving responsibilities [25–27]. Social structures commonly place women in primary caregiving roles, even when they are living with chronic illness. This has been associated with increased psychological strain and emotional burden [28]. Hemodialysis may limit a women’s ability to meet these expectations, potentially contributing to feelings of guilt and psychological distress. Cultural values that focus on marriage and fertility may further intensify the emotional burden experienced by women with chronic kidney disease in the region. In addition, mental health problems remain under-recognized and reduced access to formal psychological support may further contribute to poorer mental wellbeing among women on dialysis. These factors are not adequately captured by standard demographic or clinical variables but may exert a substantial impact on patient-reported mental health outcomes [29–31]. These contextual and gender-related psychosocial factors provide a plausible explanation for the observed disparities in mental health–related quality of life among women receiving hemodialysis. Interventions addressing mental health support, social role strain, and cultural expectations may be particularly impactful in improving overall quality of life among women on dialysis.

### Comparison with existing literature

The absence of a strong association between biochemical variables and PCS or MCS aligns with previous studies demonstrating that clinical parameters explain only a limited proportion of variance in HRQOL scores [32]. Although haemoglobin showed a borderline association with PCS, it did not reach statistical significance. Similar gender disparities in both physical and mental component scores have been reported by Lalo et al [33], whereas studies from high-income settings, such as the Netherlands, have reported higher emotional well-being among women, potentially related to higher educational attainment and social support [34]. PCS scores in the present study were substantially lower than those reported by the DOPPS across ethnicities [35], highlighting potential contextual differences. Other Egyptian studies have reported higher physical functioning scores [36] which might be due to regional or programmatic variation in patient experience.Both males and females reported similar perceptions regarding the impact and burden of kidney disease, as well as comparable scores in other quality of life domains such as cognitive function, social interaction, sleep and social support. Similar findings were observed by Cohen et al, were there was no difference between males and females in any of the 5 subscales (differences in mean score for each were <3 points) [37]. Another study in Bangladesh comparing these subdomains found that males had a higher score in EKD only suggesting male patients may perceive effects of kidney disease differently or have different coping mechanisms [38]. In the sexual function domain males reporting slightly higher mean scores which may reflect gender-related differences in perceptions or experiences of sexual wellbeing among patients with kidney disease, which could be further explored in future research.

This study provides original evidence on gender differences in quality of life among hemodialysis patients, addressing an important gap in a region where data on patient reported outcomes are limited. The large sample size and high survey completion rate strengthen the findings, and the integration of clinical and biochemical data with quality of life measures offer a robust assessment. The lack of data on some biological and treatment-related factors, including menopausal status, should be considered when interpreting the results.

## Conclusion

The persistence of lower mental health related quality of life among women despite comparable dialysis adequacy and biochemical control emphasizes the need for gender-sensitive, psychosocially informed care models. Interventions addressing mental health support, social role strain, and cultural expectations may be particularly impactful in improving overall quality of life among women on dialysis.

## Data Availability

The datasets used and/or analysed during the current study are available from the corresponding author on reasonable request.

## Abbreviations

AVF: Arteriovenous fistula
BKD: Burden of kidney disease
BMI: Body mass index
CKD: Chronic Kidney disease
CVC: Central Venous Catheter
EKD: Effects of Kidney Disease
GLM: Generalised linear model
HRQOL: Health-related quality of life
KDQOL-SF™: 1.3 Kidney disease quality of life- short form Permcath Permanent catheter
PTH: Parathyroid hormone
PCS: Physical component summary
MCS: Mental component Summary
SPKD: Symptoms and Problems of Kidney Disease
TIBC: Total iron binding capacity
TSAT: Transferrin saturation
